# Assessment of HIV molecular surveillance capacity in the European Union, 2016

**DOI:** 10.2807/1560-7917.ES.2017.22.49.17-00269

**Published:** 2017-12-07

**Authors:** Patrick Keating, Anastasia Pharris, Katrin Leitmeyer, Stefania De Angelis, Annemarie Wensing, Andrew J Amato-Gauci, Eeva Broberg

**Affiliations:** 1 Österreichische Agentur für Gesundheit und Ernährungssicherheit (AGES), Vienna, Austria; 2 European Programme for Intervention Epidemiology Training (EPIET), European Centre for Disease Prevention and Control (ECDC), Stockholm, Sweden; 3 European Centre for Disease Prevention and Control (ECDC), Stockholm, Sweden; 4 SPREAD/ESAR Programme, Luxembourg, Luxembourg

**Keywords:** HIV, drug resistance, Surveillance, European Union

## Abstract

Expanding access to HIV antiretroviral treatment is expected to decrease HIV incidence and acquired immunodeficiency syndrome (AIDS) mortality. However, this may also result in increased HIV drug resistance (DR). Better monitoring and surveillance of HIV DR is required to inform treatment regimens and maintain the long term effectiveness of antiretroviral drugs. As there is currently no formal European Union (EU)-wide collection of HIV DR data, this study aimed to assess the current HIV molecular surveillance capacity in EU/European Economic Area (EEA) countries in order to inform the planning of HIV DR monitoring at EU level. **Methods:** Thirty EU/EEA countries were invited to participate in a survey on HIV molecular surveillance capacity, which also included laboratory aspects. **Results:** Among 21 responding countries, 13 reported using HIV sequence data (subtype and/or DR) for surveillance purposes at national level. Of those, nine stated that clinical, epidemiological and sequence data were routinely linked for analysis. **Discussion/conclusion**: We identified similarities between existing HIV molecular surveillance systems, but also found important challenges including human resources, data ownership and legal issues that would need to be addressed.****Information on capacities should allow better planning of the phased introduction of HIV DR surveillance at EU/EEA level.

## Introduction

The World Health Organization’s (WHO) ‘Treat all’ recommendation and the Joint United Nations Programmes on HIV/acquired immunodeficiency syndrome (AIDS) (UNAIDS) 90–90–90 strategy are clear demonstrations of the global commitment to end the HIV/AIDS epidemic by 2030 [1,2]. In 2015, UNAIDS estimated that 46% of people living with HIV globally were on antiretroviral therapy [3]. WHO also recommends providing pre-exposure prophylaxis (PrEP) to those at substantial risk of HIV infection [4]. These initiatives should lead to a reduction in HIV incidence and AIDS mortality, but greater access to treatment without complete adherence might also result in an increase in HIV drug resistance (DR) [5].

Aside from consistent adherence to the treatment regimen, HIV DR is influenced by a number of other factors, including HIV subtype, with certain subtypes showing more rapid onset of DR than others [6]. HIV DR mutations arise from genetic alterations caused by the error-prone HIV reverse transcriptase, which may then reduce the ability of specific drugs/classes of drugs to block replication of HIV [7-9]. HIV DR mutations are classified as either transmitted (TDR), when DR occurs in a combination antiretroviral treatment (cART, a combination of two or more different classes of antiretroviral drugs)-naïve HIV-infected individual, acquired (ADR), when DR is found in cART-experienced HIV-infected individuals, or pre-treatment (PDR), when resistance is detected in individuals starting cART, that was either transmitted or acquired due to a previous antiretroviral drug exposure [10]. 

Global surveillance of HIV DR was initiated by WHO in 2004 in order to monitor emergence of HIV DR, as access to cART was scaled up worldwide. Gupta et al. reported that over the period 2004–2010, prevalence of HIV DR among treatment-naive individuals greatly increased, particularly in southern and eastern Africa, at an estimated annual incremental increase of 14% and 29%, respectively [5]. It is estimated by 2030 that if levels of PDR exceed 10% in sub-Saharan Africa, 890,000 AIDS deaths and 450,000 new HIV infections will be attributable to HIV DR [11]. Moreover, cART costs attributable to HIV DR could reach USD 6.5 billion. The WHO has led the development of the Global Action Plan on HIV DR (2017–2021) which is a call for action to all stakeholders to monitor, prevent and respond to HIV DR [10]. WHO recommends that every national AIDS programme should have a robust HIV DR surveillance and monitoring strategy [12].

HIV DR also poses a public health challenge in the European Union (EU) and European Economic area (EEA). Reports from Strategy to Control SPREAD of HIV Drug Resistance (SPREAD), an EU-funded project that collected HIV DR data across Europe from 2002 to 2008 (and later years without funding), showed that approximately one in 10 patients with newly diagnosed HIV had a transmitted DR mutation [13,14]. Changes in HIV resistance over time have been reported to both influence the choice of antiretrovirals (which include the following drug classes: protease and integrase inhibitors as well as nucleotide and non-nucleotide reverse transcriptase inhibitors) by clinicians and reflect their use [15]. Changes in antiretroviral use are primarily based on resistance testing, rather than on surveillance data. Frentz et al. demonstrated that, over the period 2002 to 2007, there was a significant decline in the prevalence of resistance mutations against protease inhibitors while the prevalence of resistance to non-nucleoside reverse transcriptase inhibitors doubled [13]. These significant changes over time in the prevalence of drug class-specific resistance mutations highlight the need for surveillance of HIV DR at the EU/EEA level to ensure the long-term effectiveness of antiretroviral drugs.

In the 2016 update to the European Centre for Disease Prevention and Control (ECDC)’s roadmap for integration of molecular typing into European-level surveillance and epidemic preparedness, HIV was highlighted as a pathogen for which more evidence on the challenges and opportunities of implementing surveillance based on molecular typing data was required before establishing a common approach to monitor HIV DR in Europe [16]. HIV DR analysis is based on genotypic and/or phenotypic testing and therefore molecular typing data are required for DR surveillance [17]. In order to inform the feasibility of and the most appropriate approach to implement an EU-level surveillance of HIV DR, we conducted a survey to assess the laboratory capacity and needs regarding molecular surveillance of HIV in EU/EEA countries.

## Methods

28 EU countries, Iceland and Norway (EU/EEA) were invited to participate in an online survey (created with EUSurvey tool [18]) on HIV molecular surveillance capacity in July 2016. As this survey investigated both surveillance systems and laboratory elements, National Focal Points for HIV/AIDS, sexually transmitted infections and hepatitis B/C as well as the National Focal Points for microbiology were invited to collaborate and complete the questionnaire. Questions covered sampling strategy, use of HIV sequence data at national level, HIV surveillance indicators, methods for HIV antiviral resistance testing, and whether national reports are produced. Reminder emails were sent to non-responders and data submission was accepted until September 2016.

The surveillance section included questions on the purpose of HIV sequence data collection, criteria used for inclusion and exclusion of cases in national HIV molecular surveillance systems, surveillance indicators used and on how and to whom surveillance data were reported. The laboratory section addressed areas including methods used and the management and reporting of laboratory data. The final section of the survey included questions on resources required by countries to perform HIV sequence analysis and to regularly report HIV DR and subtype data, as well as on potential obstacles to setting up an EU-wide surveillance system.

Counts and proportions of country responses to survey questions were calculated using R version 3.2.4, R Foundation for Statistical Learning, Vienna, Austria. Maps were generated using ECDC Map Maker (EMMa) [19]. Survey data were supplemented with HIV surveillance data submitted to the European Surveillance System (TESSy) for 2015 [20].

The results of the survey were discussed at an ECDC expert consultation meeting 27–28 October 2016, which was attended by HIV surveillance and DR experts from the EU, Switzerland and the WHO.

## Results

A total of 21 of 30 EU/EEA countries responded to this survey. An overview of the uses of HIV sequence data at national level across the EU can be seen in the Figure. About two thirds of responding countries (n = 13 countries) reported using HIV sequence data for surveillance purposes. Nineteen countries reported that HIV sequence data are used at national level for monitoring of HIV DR. In addition, 19 and 15 countries use HIV sequence data at national level for assessment of HIV subtype and phylogenetic analysis of transmission events respectively.

**Figure:**
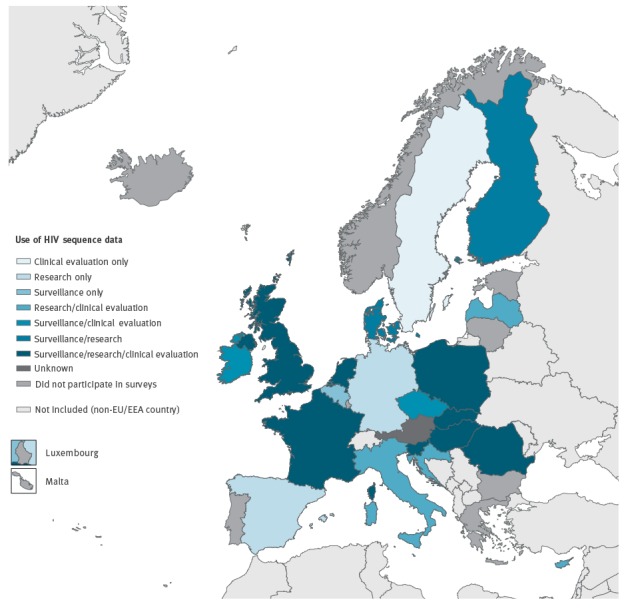
Main uses of HIV sequence data in European Union countries, 2016 (n = 21)

EEA: European Economic area; EU: European Union.

Administrative boundaries: EuroGeographics, UN-FAO.

A summary of the survey findings on the key elements of HIV molecular surveillance and reporting in the EU is presented in Table 1. Differences were found in the patients selected for sequence-based characterisation of HIV. Of 19 countries that reported using HIV sequence data at national level to monitor HIV DR, 12 countries obtain samples for sequence-based characterisation from cART-naïve individuals newly diagnosed with HIV, nine countries obtain samples from both cART-naïve individuals newly diagnosed with HIV and cART-experienced individuals with detectable HIV load. Seven countries performed longitudinal collection (collection of multiple sequences per individual), six cross-sectional collection (collection of one sequence per individual) of HIV sequences, and an additional two countries reported cross-sectional and longitudinal sequence collection. Twelve countries were able to estimate the national coverage of HIV sequence data, which ranged from 15 to 100% of individuals who met the case definition of HIV.

**Table 1:** Overview of molecular surveillance of HIV in European Union countries, 2016 (n = 21)

Country	Collect HIV sequence data and use for surveillance at national level	Patients selected for sequence-based characterisation in clinical practice	Collection of HIV sequences	Purpose of HIV sequence data	National coverage of HIV sequence data (%)	Data linkage	Frequency of reporting of sequence data	National HIV DR report	Submission to SPREAD
Austria	No	Majority of HIV patients	Not performed	Not applicable	No data	No, aggregated^a^	NA	No	Yes
Belgium	Yes	cART-naive newly HIV diagnosed^b^ and cART-experienced with HIV^c^	Cross-sectional^d^	DR^e^/subtype^f^	No data	No, aggregated^a^	Real-time	Yes	Yes
Croatia	No	Selected patient groups (e.g. MSM recently infected abroad) and in all treatment failures	Longitudinal^h^	DR^e^/subtype^f^/transmission^g^	15	Complete or full data linkage^i^	NA	No	Yes
Cyprus	No	cART-naive newly HIV diagnosed^b^ and cART-experienced with HIV^c^	Cross-sectional^d^	DR^e^/subtype^f^/transmission^g^	100	Complete or full data linkage^i^	Annually	No	Yes
Czech Republic	Yes	cART-naive newly HIV diagnosed^b^ and cART-experienced with HIV^c ^and upon request of a physician	Continually; one sequence per individual	DR^e^/subtype^f^	100	Complete or full data linkage^i^	Real-time	No	Yes
Denmark	Yes	Ca. 70% of cART-naive newly HIV diagnosed^b^ and a large proportion of cART-experienced with HIV^c^	Cross-sectional^d^	DR^e^/subtype^f^/transmission^g^	70	Virological and epidemiological information can be linked	Annually	Yes	Yes
Finland	Yes	cART-naive newly HIV diagnosed^b^ and cART-experienced with HIV^c ^and cART-naïve initiating first line cART^j^ and upon request of a physician	Cross sectional^d^ and Longitudinal^h^	DR^e^/subtype^f^/transmission^g^	80	Complete or full data linkage^i^	Real-time	No	Yes
France	Yes	cART-naive newly HIV diagnosed^b^ and cART-experienced with HIV^c^ and cART-naïve initiating first line cART^j^ and pregnant women and source patients with a detectable HIV load	Cross-sectional^d^	DR^e^/subtype^f^/transmission^g^	No data	Complete or full data linkage^i^	Annually	Yes	No data
Germany	No	cART-experienced with HIV^c^ and cART-naïve initiating first line cART^j^	Cross-sectional^d^ and longitudinal^h^	DR^e^/subtype^f^/transmission^g^	No data	Different database variables are linked^k^	Real-time	No	No
Hungary	Yes	cART-naive newly HIV diagnosed^b^ and cART-naïve initiating first line cART^j^	Longitudinal^h^	DR^e^/subtype^f^/transmission^g^	No data	Complete or full data linkage^i^	Monthly	Yes	No
Ireland	Yes	cART-naive newly HIV diagnosed^b^ and cART-naïve initiating first line cART^j^ and those with detectable viral load where a regimen change is considered	Longitudinal^h^	DR^e^/subtype^f^/transmission^g^	65	Complete or full data linkage^i^	Annually	Yes	No
Italy	No	Proportion of cART-experienced with HIV^c^	Not applicable	DR^e^/subtype^f^/transmission^g^	No data	No, aggregated^a^	NA	No	No data
Latvia	No	cART-naïve initiating first line cART^j^	Not applicable	DR^e^/subtype^f^	No data	No, aggregated^a^	Real-time	No	No
Netherlands	Yes	cART-naive newly HIV diagnosed^b^ and cART-naïve initiating first line cART^j^	No data	DR^e^/subtype^f^/transmission^g^	35	Complete or full data linkage^i^	Annually	Yes	Yes
Poland	Yes	cART-experienced with HIV^c ^and cART-naïve initiating first line cART^j^	Longitudinal^h^	DR^e^/subtype^f^/transmission^g^	40	Partial data submitted to SPREAD programme; otherwise data are not linked	Annually	No	Yes
Romania	Yes	Among some patients with virologic failure	Longitudinal^h^	DR^e^/subtype^f^/transmission^g^	No data	No, aggregated^a^	NA	No	No
Slovakia	Yes	50% of each of the following: cART-naive newly HIV diagnosed^b^ and cART-experienced with HIV^c^ and cART-naïve initiating first line cART^j^ and also individuals with ART failure	Cross-sectional^d^	DR^e^/subtype^f^/transmission^g^	50	Different database variables are linked^k^	Real-time	No	Yes
Slovenia	Yes	cART-experienced with HIV^c^	Cross-sectional^d^	DR^e^/subtype^f^/transmission^g^	50	Complete or full data linkage^i^	Annually	Yes	Yes
Spain	No	cART-naive newly HIV diagnosed^b^ and cART-naïve initiating first line cART^j^	Cross-sectional^d^	Not applicable	No data	Not applicable	NA	No	No data
Sweden	No	cART-naive newly HIV diagnosed^b^ and cART-experienced with HIV^c^ and cART-naïve initiating first line cART^j^	Longitudinal^h^	DR^e^/subtype^f^	65	Data are submitted on a case by case level but not linked	For clinical purpose mainly	Yes	No data
United Kingdom	Yes	cART-naive newly HIV diagnosed^b^ and cART-experienced with HIV^c^ and cART-naïve initiating first line cART^j^	Longitudinal^h^	DR^e^/subtype^f^/transmission^g^	70	Different database variables are linked^k^	Annually	Yes	No data

cART: combination antiretroviral therapy; DR: drug resistance; MSM: men who have sex with men; NA: not available; SPREAD: Strategy to Control SPREAD of HIV Drug Resistance.

^a^ Data are not linked and are available on an aggregated level only.

^b^ All cART-naive individuals newly diagnosed with HIV.

^c^ All cART-experienced individuals with a detectable HIV load.

^d^ Only one sequence per individual is collected.

^e^ Monitoring HIV DR.

^f^ Assess HIV subtypes.

^g^ Conduct phylogenetic analysis of transmission events.

^h^ Longitudinal in a cohort; multiple sequences per individual can be collected.

^i ^Integrated clinical, epidemiological and virological data are submitted on a case-by-case level.

^j ^All cART naïve individuals initiating first line cART.

^k^ Clinical, epidemiological and laboratory variables from different databases are linked.

Among 13 countries reporting using HIV sequence data for surveillance (subtype and/or DR), nine stated that linkage between clinical, epidemiological and sequence data with one or more databases took place (Table 1). Of those, seven countries reported integration of clinical, epidemiological and virological data at case level. 

Moreover, among the 13 using HIV sequence data for surveillance, seven countries stated that they report sequence data annually, four in real time, one monthly and one did not address this question. SPREAD was the most commonly reported project to which countries performing surveillance of HIV sequences submitted HIV DR data (n = 8 countries). Eight of 13 countries performing molecular surveillance of HIV stated that they produced a national HIV DR report.

### Surveillance system

Of 13 countries performing molecular surveillance of HIV, 11 stated that they employed a number of different strategies for the surveillance of HIV sequence data at national level. Eleven countries reported that they perform resistance testing based on samples routinely generated in clinical practice. Specific cohorts/projects were the second most highly reported sample source (n = 6 countries). Seven countries stated that they believed that their sampling strategy was fully representative for the national HIV epidemic. Three countries reported that sampling was representative for specific regions. No country stated that they used a specific sample size calculation method for estimating the prevalence of DR at national level for extrapolating the measured prevalence to estimate the true population prevalence (number of cases).

As seen in Table 2, 18 of 21 countries participating in the current study, were able to provide an estimate of the numbers of sequences collected at national level in 2015, with answers ranging from less than 100 sequences to more than 2,000. Comparison of these estimates of HIV sequence data with HIV surveillance data submitted to TESSy in 2015, and assuming one sequence was collected per newly diagnosed individual, revealed country-specific proportions of new HIV diagnoses with sequence data ranging from 10 to 100%.

**Table 2:** Estimated proportion of new HIV diagnoses in European Union countries with sequence data, 2015 (n = 21)

Country	Estimated number of HIV sequences collected in 2015^a^	Number of new HIV diagnoses reported in 2015^b^	Estimated % of new HIV diagnoses with sequence data
Austria	No data	264	Not calculable
Belgium	Between 500 and 999	1,001	75
Croatia	Less than 100	117	43
Cyprus	No data	80	Not calculable
Czech Republic	Between 100 and 199	266	56
Denmark	Between 100 and 199	277	54
Finland	Between 100 and 199	174	86
France	Between 500 and 999	3,943	19
Germany	Over 2,000	3,674	Not calculable
Hungary	Less than 100	271	19^c^
Ireland	Between 500 and 999	486	65^c^
Italy	Between 200 and 499	3,444	10
Latvia	Between 100 and 199	393	38
Netherlands	Between 200 and 499	802	44
Poland	Between 200 and 499	1,275	27
Romania	Between 100 and 199	756	20
Slovakia	Between 100 and 199	86	100
Slovenia	Less than 100	48	50^c^
Spain	No data	3,428	Not calculable
Sweden	Between 200 and 499	447	78
United Kingdom	Over 2,000	6,078	Not calculable

^a^ The midpoint of the range was used to estimate the proportion of new HIV diagnoses with sequence data.

^b^ Data were obtained from TESSy [20].

^c ^Country directly provided % of new HIV diagnoses with sequence data.

Of the 13 countries performing molecular surveillance of HIV, ten stated that surveillance indicators are used for HIV molecular surveillance. The most commonly reported indicators were (i) the proportion of sequences available among all newly reported HIV cases in a given year (n = 10 countries), (ii) the proportion of non-B subtype sequences among all reported sequences in a given year (n = 10 countries), and (iii) the proportion of available sequences with relevant resistance mutations according to the Stanford HIV Drug resistance (n = 9 countries). The most frequently reported inclusion criteria for HIV molecular surveillance were: cART-naïve individuals newly diagnosed with HIV (n = 6 countries) and cART-experienced individuals with detectable HIV load (n = 4 countries). Samples without known HIV RNA level (n = 7 countries) and an HIV viral load of less than 1,000 copies/mL (n = 5 countries) were reported as the primary exclusion criteria for not collecting HIV sequences. In terms of collection of HIV sequence data, nine countries reported continuous collection of generated sequences and three reported continuous collection of samples with retrospective batch-wise sequence analysis.

### Laboratory

Based on the survey, the most frequently reported specimens taken for HIV sequence analysis among countries performing HIV molecular surveillance (n = 13 countries) were ethylenediaminetetraacetic acid (EDTA)-plasma (n = 10 countries). All countries used population sequence analysis to generate their sequence data. Three countries also reported using next-generation sequencing (NGS) analysis to generate their HIV sequence data. The Rega HIV subtyping tool (n = 6 countries) and the Stanford HIV Drug resistance database (n = 3 countries) were the most widely reported tools for subtype analysis. There was little difference among countries in the genes used for HIV genome sequencing, with protease (n = 13 countries), reverse transcriptase (n = 13 countries) and integrase (n = 11 countries) being the most commonly reported genes sequenced.

Ten countries performing molecular surveillance of HIV stated that laboratories report HIV sequences. Eleven countries stated that HIV sequence data were reported at the nucleotide level and one at amino acid level. The primary database types used for storage of HIV sequence data were Excel (n = 3 countries), SQL (n = 2 countries) and Access (n = 2 countries).

### Resources and obstacles

Within the 21 countries participating in the current study, a change of guidelines/policy (n = 10 countries) and additional personnel (n = 10 countries) were highlighted as key resources needed to implement HIV sequence analysis. No further explanation was provided by any country on the type of guideline/policy changes needed to implement HIV sequence analysis. Database solutions (n = 8 countries), data entry capacity and data transfer (n = 6 countries) and support in data analysis (n = 5 countries) were the main types of technical support required for the implementation of regular reporting of HIV DR data. Similar results were found for the reporting of HIV subtype data. Among the 12 countries that do not currently report HIV DR at national level, database solutions (n = 5 countries) and support in data analysis (n = 5 countries) were the most frequently highlighted technical support requirements.

There was no clear preferred reporting format for HIV DR in the EU. Aggregated reporting of DR prevalence data per drug (n = 9 countries) or case- and (nucleotide level) sequence-based reporting (n = 7 countries) were both similarly preferred formats. Respondents disagreed on the most appropriate strategy to implement molecular surveillance of HIV in countries without existing HIV molecular surveillance (n = 8 countries). Two of these eight countries supported periodic surveys on pre-treatment or acquired resistance, one country supported sentinel sampling based on agreed standards and indicators and another supported comprehensive collection of case-based HIV resistance data. No country elaborated further on their preferred strategy to implement molecular surveillance of HIV. Among the 21 countries participating, a number of obstacles to sharing national sequence data at international level were highlighted: human resources (n = 13 countries), database compatibility (n = 13 countries), data ownership (n = 13 countries), ethical (n = 9 countries) and legal issues (n = 9 countries) were the most frequently reported.

## Discussion

The results of this survey provide an overview of the current status of HIV molecular surveillance in the EU. Thirteen of the 21 responding countries reported that they are already using sequence data for the surveillance of HIV to some extent, with a number of countries also submitting HIV sequence data to the SPREAD project and/or including HIV DR data in national surveillance reports. Several differences between national HIV molecular surveillance systems were noted, including criteria used for inclusion and exclusion of cases in national HIV molecular surveillance systems, however, similarities between systems were also found (e.g. data linkage, populations under surveillance and laboratory methods used). Knowledge of these similarities and differences will be used to inform a pilot study on HIV DR surveillance in the EU.

This study also identified some similarities in the needs of EU countries for performing and regularly reporting HIV sequence data, as well as in the opinions on the obstacles for submitting national sequence data to the international level among participating countries. Human resources, data management, legal and ethical issues could pose significant challenges to establishing an EU-wide molecular surveillance system. However, these issues are not perceived to be absolute obstacles to a phased implementation of a HIV molecular surveillance system, where countries could submit data as and when it became feasible for them to do so. The Centers for Disease Prevention and Control (CDC) in the United States (US) have similarly highlighted that legislative issues need to be considered in relation to HIV DR surveillance, such as review and potentially revision of State or local HIV laws and regulations to ensure reporting of HIV nucleotide sequence data at national level [21]. The WHO Global Action Plan on HIV drug resistance highlighted similar challenges faced by countries when implementing HIV DR surveillance, including issues related to data ownership, laboratory capacity, human resources, competing public health priorities and national regulations [10].

Across EU countries, considerable variation was found in the estimated proportion of new HIV diagnoses for which sequence data were available. These differences may reflect the sampling strategies applied to surveillance of HIV sequence data across the EU. However, these estimates must be considered with caution, as countries provided a range for the estimate of the number of HIV sequences collected and the midpoint of this range was used for calculation purposes. Moreover longitudinal sampling was not taken into account.

The SPREAD project has performed HIV DR surveillance in Europe for more than 10 years, and has shown that TDR prevalence has stabilised at ca 8%, from 2002 to 2010, while also reporting an increase in resistance to specific drug classes [13,14,22]. A meta-analysis by Rhee et al. reported a similar overall TDR prevalence of 9.4% in Europe between 2000 and 2013 [23]. Individual country reports from Belgium 2014, Ireland 2015 and France 2009 have shown a similar stabilising of the prevalence of TDR over time [24-26]. However, analyses from Switzerland have shown fluctuation in TDR prevalence between 1995 and 2012, with reductions over time likely due to the introduction of new drug classes [27]. The Swiss study emphasised the need for continuous development and widespread distribution of new drugs in order to reduce TDR prevalence. Furthermore, analysis of HIV DR surveillance data in the Netherlands allowed researchers to better understand transmission clusters among men who have sex with men (MSM) [28]. They identified self-sustaining HIV clusters that were present since the 1990s and emphasised the need for intensified efforts to prevent further infections.

### Weaknesses and limitations

The results of this study need to be considered in light of the survey’s limitations. Firstly, the overall response rate was 21/30, which may have been negatively influenced by the timing of the survey (during the summer period). It is more likely that those countries with something positive to report in this field would have responded, leading to a slight overestimation of the true proportions in the EU. The results do not therefore provide a full understanding of HIV molecular surveillance capacity in the EU. Secondly, the accuracy of the content is very much dependent on who has been tasked to fill in the data and there may be some variability due to incomplete knowledge of the situation by the respondent. Thirdly, only rough estimates of the proportion of HIV cases with sequence data could be calculated due to the nature of the data available. Finally, caution is needed when interpreting the data on HIV molecular surveillance capacity, as countries performing HIV molecular surveillance represent just under half of the total number of EU countries and no data on HIV molecular surveillance capacity were obtained from seven EU or any EEA countries.

### The way forward

The results of this survey were presented and discussed at an ECDC expert consultation meeting. It was broadly agreed that ECDC could have an important role in establishing a formal HIV DR surveillance system, through standardisation of the reporting procedure and by helping reduce some of the reporting issues identified in the survey. However, more structural barriers to performing HIV DR surveillance including human resources, ethical considerations and changes in guidelines/policy could only be addressed at a national level. The objectives for HIV DR monitoring at EU/EEA level, and the scope and format of an EU/EEA surveillance system for HIV DR monitoring were key items discussed at this meeting. Estimating TDR prevalence among newly diagnosed individuals was generally supported as a primary objective for an EU/EEA-wide HIV DR system, which is similar to that of the US CDC’s Molecular HIV Surveillance (MHS) system and Australia’s HIV DR surveillance reporting [21,29]. There was a general consensus that a pilot study on collection of HIV DR data should be conducted, which should focus on a limited number of countries that already collect HIV DR data and are able to link it to demographic data. The pilot study will serve to explore the feasibility of standardising reporting, an agreement for a European surveillance protocol and evaluation of representativeness of the studied population. The outcomes of the pilot study will be discussed within the European HIV surveillance network and establishment of European HIV DR surveillance will be agreed with the countries that can adjust their national policies. Currently, HIV DR surveillance is not planned to be mandatory for EU countries.

In conclusion, this survey has identified some capacity for HIV DR surveillance in the EU, but also important challenges that will take time to address. The next steps will involve the conduct of a pilot study of HIV DR surveillance in selected EU countries. The phased implementation of a HIV DR surveillance system in the EU/EEA should allow better data for monitoring of HIV DR over time, by drug class, among risk groups and by geographic location. This will allow for a better understanding of HIV DR, which is of primary concern for public health since it has the potential to impact the success of first-line cART regimens, resulting both in potentially negative patient outcomes and the potentially higher cART treatment costs associated with second or third-line regimens.
